# The Safety of Two Different Oral Misoprostol Dosing Strategies for Labor Preinduction at Term: A Single-Center Retrospective Cohort Study

**DOI:** 10.3390/jcm15062425

**Published:** 2026-03-22

**Authors:** Magdalena Adamczyk, Witold Włodzimierz Kędzia, Julia Rogalska, Paulina Mularczyk, Małgorzata Kędzia

**Affiliations:** 1 Department of Reproduction and Gynecology, Poznan University of Medical Sciences, 60-535 Poznan, Poland; mal.gin@poczta.fm; 2 Student Scientific Association of Department of Reproduction and Gynecology, Poznan University of Medical Sciences, 60-535 Poznan, Poland; witek3214@gmail.com (W.W.K.); juliarogalska231@gmail.com (J.R.); paulinamularczyk2002@gmail.com (P.M.)

**Keywords:** misoprostol, labor preinduction, term pregnancy, cervical ripening, oral prostaglandin

## Abstract

**Background/Objectives:** Preinduction of labor is commonly performed in women with unfavorable cervical conditions at term. Oral misoprostol is increasingly used due to its ease of administration and effectiveness; however, optimal dosing regimens remain under investigation. This study aimed to evaluate the safety and effectiveness of two oral misoprostol regimens (25 µg every 2 h versus 50 µg every 4 h) for preinduction of labor in term pregnancies. **Methods:** This single-center retrospective cohort study included 270 women with singleton term pregnancies who underwent oral misoprostol preinduction. Women received either 25 µg every 2 h (*n* = 60) or 50 µg every 4 h (*n* = 210) according to routine clinical protocols. Data were collected from electronic medical records and included demographic and obstetric characteristics, labor course, need for additional interventions (e.g., Foley catheter), and neonatal outcomes. The primary outcome was a composite maternal and neonatal safety endpoint. Secondary outcomes included mode of delivery, need for Foley catheter use, and time to delivery. **Results:** Both regimens were effective in facilitating labor progression. In crude analysis, the need for additional cervical ripening with a Foley catheter was higher in the 50 µg group compared with the 25 µg group (37.1% vs. 21.7%, *p* = 0.037); however, after stratification by prelabor rupture of membranes (PROM), this difference was no longer statistically significant (*p* = 0.39). Cesarean section rates did not differ significantly between groups (29.0% vs. 20.0%, *p* = 0.22). The time from the last misoprostol dose to delivery was shorter in the 50 µg group, but the difference was not statistically significant (*p* = 0.17). Neonatal outcomes, including birthweight, Apgar scores, and umbilical cord blood gas parameters, were comparable between groups. No severe maternal or neonatal adverse events were recorded. **Conclusions:** In this single-center retrospective cohort study, the 25 μg every 2 h and 50 μg every 4 h oral misoprostol regimens were associated with comparable obstetric and neonatal outcomes within the analyzed parameters. No significant differences in recorded maternal or neonatal safety outcomes were observed. Selection of the dosing regimen should take into account individual clinical factors, including parity, cervical status, and membrane status.

## 1. Introduction

Induction of labor is a common obstetric intervention, performed in approximately 20–30% of term pregnancies worldwide, most frequently due to maternal or fetal indications such as diabetes, hypertensive disorders of pregnancy, post-term gestation, or prelabor rupture of membranes (PROM) [1,2,3]. When the cervix is unfavorable, cervical ripening is recommended to improve the likelihood of vaginal delivery, shorten labor duration, and reduce the risk of failed induction and cesarean delivery [4,5]. Misoprostol, a synthetic prostaglandin E1 analogue, has been widely used for cervical ripening and induction of labor because of its proven efficacy, low cost, stability at room temperature, and ease of administration [6,7,8,9]. Although initially used off-label, misoprostol is now recommended by international guidelines, including those of the World Health Organization (WHO), for labor induction at term when administered in low oral doses [10]. Oral administration of misoprostol has been shown to be associated with a lower risk of uterine tachysystole and hyperstimulation compared with vaginal administration, while maintaining a favorable balance between efficacy and safety [11,12,13,14].

Angusta^®^ was introduced to provide an approved low-dose oral misoprostol formulation, allowing more precise dosing compared with 200 μg tablets (e.g., Cytotec^®^). Different low-dose strategies have been implemented in clinical practice, aiming to balance efficacy with maternal and neonatal safety [15]. The most commonly used regimens include 25 μg administered every 2 h and 50 μg administered every 4 h. Although oral misoprostol is widely used for cervical ripening and labor induction, the optimal dosing regimen remains a matter of debate.

Several retrospective cohort studies have recently evaluated the effectiveness and safety of different oral misoprostol dosing regimens in routine clinical practice. Bendix et al. compared high- and low-dose oral misoprostol protocols in a Danish retrospective cohort and demonstrated comparable effectiveness, while highlighting differences in induction dynamics and intervention rates [16]. Wesselius et al. reported improved neonatal outcomes following induction with 25 μg compared with 50 μg oral misoprostol [17]. Similarly, Kehl et al. and Fathi Roodsari et al. analyzed low- versus higher-dose oral misoprostol regimens and confirmed acceptable safety profiles, although obstetric outcomes varied depending on dosing strategy and clinical context [18,19]. Despite these important contributions, direct comparisons of the specific 25 μg every 2 h versus 50 μg every 4 h regimens remain relatively limited, particularly within standardized institutional protocols and retrospective single-center cohort studies.

Several maternal factors have been shown to influence the success of labor induction, including parity and maternal body mass index (BMI). Nulliparity is a well-established risk factor for prolonged labor and increased rates of cesarean delivery following induction [13,20]. In contrast, the role of maternal BMI in induction outcomes remains controversial, with some studies suggesting reduced efficacy in obese women, while others report no significant impact on induction success or safety [21,22,23,24].

In March 2025, oral preinduction of labor with Angusta^®^ was implemented as a standard procedure at the Gynecological Obstetric Clinical Hospital of Poznan University of Medical Sciences, Poland. The protocol included oral misoprostol preinduction in term singleton pregnancies with an unfavorable cervix, with a maximum cumulative dose not exceeding 200 micrograms, in line with international recommendations [10]. Two dosing regimens were used in routine clinical practice: 25 micrograms every 2 h and 50 micrograms every 4 h.

The primary aim of this study was to compare the safety of these two oral misoprostol preinduction regimens. Specifically, we evaluated maternal and neonatal outcomes associated with each dosing schedule in term pregnancies undergoing labor preinduction. By analyzing real-world data from a single tertiary center, this study seeks to contribute to the optimization of oral misoprostol dosing strategies and to support evidence-based clinical decision-making in labor preinduction.

## 2. Materials and Methods

### 2.1. Study Design and Setting

This retrospective cohort study was conducted at the Gynecological Obstetric Clinical Hospital of Poznan University of Medical Sciences, Poland, and covered the period from 1 March to 1 November 2025, corresponding to the introduction of oral misoprostol (Angusta^®^) for labor preinduction in our institution. The study was designed and reported in accordance with the RECORD guidelines for observational studies using routinely collected health data [25].

Clinical data were obtained from the hospital’s internal electronic medical record system (Eskulap) after a detailed review of records related to the administration of Angusta^®^.

During the study period, oral misoprostol was used for labor preinduction in 282 pregnant women out of 3157 deliveries (8.9%). After exclusion of patients who did not meet protocol criteria, 270 women were included in the final analysis.

This retrospective observational study used anonymized routinely collected clinical data and did not involve any intervention beyond standard clinical care. According to the decision issued by the Bioethics Committee of the Poznan University of Medical Sciences (KB-51/26; 21 January 2026), this study did not constitute a medical experiment, and therefore formal ethical approval was not required.

#### 2.1.1. Preinduction Protocol

Patients received either:

- 25 µg of oral misoprostol every 2 h, or
- 50 µg of oral misoprostol every 4 h,

Administered orally with a glass of water. The maximum cumulative dose, regardless of dosing regimen, was 200 µg within 24 h, in accordance with international recommendations [10].

Preinduction was discontinued when:

- regular uterine contractions and active labor began,
- uterine tachysystole or hyperstimulation occurred,
- signs of fetal compromise or threat to fetal well-being were detected.

Preinduction Assessment and Eligibility

Before initiation of oral misoprostol preinduction, each patient underwent a comprehensive safety assessment, including:

- gynecological examination with cervical assessment using the Bishop score,
- fetal ultrasound to assess fetal presentation and estimated fetal weight,
- placental localization,
- evaluation of amniotic fluid volume using the amniotic fluid index (AFI),
- Doppler flow measurements in the umbilical artery and middle cerebral artery,
- cardiotocography (CTG).

Only patients with an unfavorable cervix (Bishop score ≤ 6) were eligible for misoprostol preinduction. Bishop score was assessed at admission by a board-certified obstetrician using standardized institutional criteria.

#### 2.1.2. Induction Procedure and Clinical Management

Labor preinduction was initiated with oral misoprostol administered according to one of the two dosing regimens described above. Misoprostol was administered for a maximum of 24 h (maximum cumulative dose 200 µg). Clinical assessment, including fetal monitoring and cervical evaluation, was performed regularly during the induction process.

After pharmacological preinduction, further management depended on cervical status and uterine activity. If the Bishop score remained below 6 and regular uterine contractions were not established, mechanical cervical ripening was initiated using a Foley catheter, which was left in place for up to 24 h or until spontaneous expulsion, whichever occurred first. Conversely, when cervical dilation of at least 3 cm was achieved in the presence of regular uterine contractions, labor augmentation was continued with intravenous oxytocin. Oxytocin infusion was initiated at a starting dose of 2 mU/min and increased every 20–30 min according to uterine response, up to a maximum dose of 20 mU/min. Additional doses of misoprostol were not administered after Foley catheter placement.

Labor induction was considered unsuccessful when active labor was not achieved after completion of the institutional preinduction protocol, including pharmacological preinduction with oral misoprostol (maximum cumulative dose 200 µg within 24 h) followed by mechanical cervical ripening or oxytocin augmentation when indicated. No single predefined time threshold alone was used to define failed induction; rather, the decision was based on a lack of labor progression after completion of the stepwise induction protocol and clinical assessment of maternal and fetal conditions. In such cases, delivery was completed by cesarean section according to standard obstetric indications.

#### 2.1.3. Treatment Allocation and Clinical Decision-Making

Allocation to the oral misoprostol dosing regimen (25 µg every 2 h or 50 µg every 4 h) was not randomized. Both regimens were used as part of routine clinical practice during the study period and represented locally implemented protocols for labor preinduction. The selection of dosing schedule was made by the attending obstetrician based on individual clinical assessment, including cervical status, indication for induction, maternal clinical condition, and fetal well-being. As this was a retrospective observational study, no predefined allocation algorithm was applied, and treatment assignment was based on routine clinical judgment.

#### 2.1.4. Inclusion and Exclusion Criteria

Patients were eligible for inclusion if they met all of the following criteria:

- singleton pregnancy at term (≥37 weeks of gestation),
- cephalic fetal presentation,
- indication for labor induction according to local clinical practice,
- unfavorable cervix defined as a Bishop score ≤ 6,
- preinduction with oral misoprostol according to the institutional protocol.

Exclusion criteria included:

- history of previous cesarean section or other uterine surgery contraindicating labor induction,
- multiple pregnancy,
- contraindications to vaginal delivery,
- known fetal anomalies incompatible with vaginal birth,
- missing key clinical data required for analysis.

The most common indications for labor preinduction were:

- gestational diabetes,
- chronic or gestational hypertension,
- preeclampsia,
- prelabor rupture of membranes without spontaneous uterine contractions,
- fetal growth restriction,
- post-term pregnancy.

Additional indications included intrahepatic cholestasis of pregnancy, suspected fetal macrosomia, idiopathic polyhydramnios, oligohydramnios, and serological incompatibility.

#### 2.1.5. Monitoring and Further Management

Prior to the first dose of misoprostol, each patient underwent a 30-min CTG recording, which was continued for 60 min after drug administration. If the CTG was reassuring, subsequent monitoring was performed approximately 20 min before the next dose and continued for about 30 min.

Administration of misoprostol was discontinued when the maximum cumulative dose of 200 µg was reached, uterine hyperstimulation occurred, or active labor commenced.

After pharmacological preinduction, patients were transferred to the delivery ward for further management according to institutional protocol.

Definitions

Uterine tachysystole was defined as more than five contractions in 10 min averaged over a 30-min period. Uterine hyperstimulation was defined as tachysystole associated with non-reassuring fetal heart rate changes. Non-reassuring cardiotocography (CTG) included repetitive late decelerations, prolonged decelerations, or persistent minimal variability.

#### 2.1.6. Protocol Deviations

**Figure 1:**
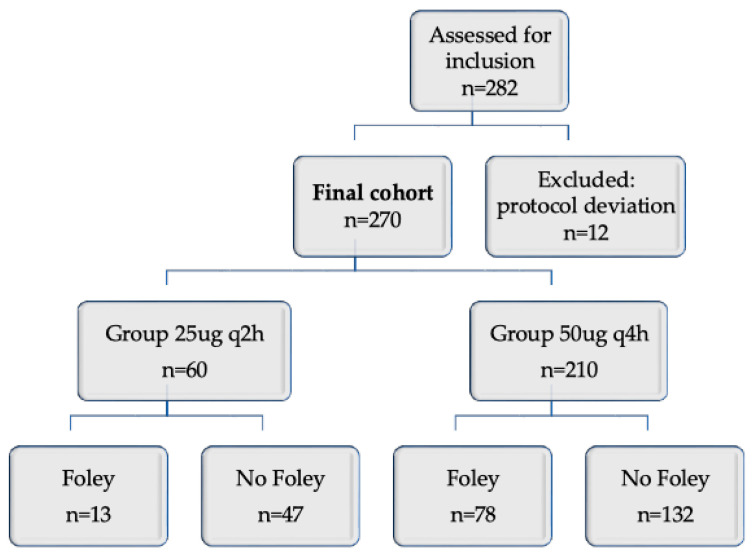
Study flowchart.

After review of all medical records, it was identified that 12 patients received a dosing regimen inconsistent with the study protocol, consisting of an initial dose of 50 µg followed by 25 µg every 2 h after 4 h. These patients were excluded from further analysis (Figure 1).

A total of 282 women underwent labor preinduction with oral misoprostol during the study period. Ten women were excluded due to protocol deviations in the dosing regimen. The final cohort consisted of 270 women, of whom 60 received oral misoprostol at a dose of 25 µg every 2 h and 210 received 50 µg every 4 h. The need for additional mechanical cervical ripening with a Foley catheter was recorded in both groups.

#### 2.1.7. Study Outcomes

The primary outcome of the study was a composite safety outcome including selected maternal and neonatal adverse events recorded in the medical documentation. Maternal safety outcomes included uterine tachysystole, uterine hyperstimulation, need for tocolytic therapy, and postpartum hemorrhage. Neonatal safety outcomes included non-reassuring cardiotocography, the need for neonatal resuscitation, transfer to the neonatal intensive care unit (NICU) and sepsis.

Secondary outcomes included mode of delivery (vaginal or cesarean), need for Foley catheter use during induction, and time from induction to delivery.

Additional safety parameters suggested in the previous literature, such as placental abruption, chorioamnionitis, meconium-stained amniotic fluid, or maternal analgesic requirements, were not systematically recorded in all cases and therefore were not included in the primary analysis. These limitations are acknowledged in the Discussion.

### 2.2. Statistical Analysis

PQStat statistical software (version 1.8.6.126, PQStat Software, Poznan, Poland) was used for statistical analysis. The Kolmogorov–Smirnov test was used to verify normality. Group comparisons were analyzed using Student’s *t*-test for variables with a normal distribution and the Mann–Whitney rank sum test for variables with a nonnormal distribution. For nonquantitative characteristics, groups were compared using the chi-square test with Yates correction. The logistic regression test was used to construct a model that determined the odds [OR] of vaginal delivery based on the studied variables. Statistical significance was defined as *p* < 0.05 (two-sided).

## 3. Results

A total of 270 women underwent labor preinduction with oral misoprostol, including 60 patients in the 25 µg group and 210 patients in the 50 µg group. Maternal age was comparable between groups (mean 30 ± 4 vs. 31 ± 5 years; *p* = 0.70). The median gestational age at delivery was 39 weeks in both groups, indicating similar pregnancy maturity at the time of preinduction.

Women in the 50 µg group had a higher median BMI compared with those in the 25 µg group (31 vs. 27); this difference reached statistical significance (*p* = 0.0001). Parity distribution did not differ significantly between groups.

A Foley catheter was inserted in 91 out of 270 women included in the final analysis, reflecting the need for additional cervical ripening following pharmacological preinduction. Foley catheter use was significantly more frequent in the 50 µg group compared with the 25 µg group (78 vs. 13 patients, *p* = 0.03).

Among women who required Foley catheter placement for mechanical cervical ripening, 26 subsequently developed spontaneous uterine contractions without the need for further pharmacologic stimulation. In 31 cases, after removal of the Foley catheter, artificial rupture of membranes was performed due to advanced cervical dilation (≥6 cm). In the remaining 34 women, labor augmentation with intravenous oxytocin was initiated following Foley catheter removal.

The mode of delivery did not differ significantly between groups. Spontaneous vaginal delivery occurred in 48 women (80.0%) in the 25 µg group and in 149 women (71.0%) in the 50 µg group, while cesarean section was performed in 12 (20.0%) and 61 (29.0%) patients, respectively. Statistical analysis using the chi-square test showed no significant association between misoprostol dose and delivery mode (χ^2^ = 1.51, *p* = 0.22), indicating that the observed differences were not statistically significant.

### 3.1. Association Between Parity and Mode of Delivery

The rate of cesarean delivery was significantly higher among primiparous women compared with multiparous women. Cesarean section was performed in 65 of 210 primiparas (31.0%) and in 9 of 60 multiparas (15.0%). The association between parity and mode of delivery was statistically significant (Pearson’s chi-square test: χ^2^ = 6.0; *p* = 0.014), which was confirmed by Fisher’s exact test (*p* < 0.05).

This association was further confirmed in logistic regression analysis, which identified parity as a significant predictor of delivery outcome (B = 0.9218; S.E. = 0.3896; Wald test *p* = 0.0178). Multiparity was associated with increased odds of vaginal delivery (OR = 2.51; 95% CI: 1.17–5.39), indicating that multiparous women had more than two-fold higher likelihood of achieving vaginal birth compared with nulliparous women. The results of the multivariable logistic regression analysis are presented in Table 1.

### 3.2. Association Between Foley Catheter Use and Parity

The use of a Foley catheter was comparable between multiparous and primiparous women. The association between parity and Foley catheter use was assessed using Pearson’s chi-square test, which showed no statistically significant difference between the groups (χ^2^ = 0.005; *p* = 0.8948). This finding was confirmed by Fisher’s exact test (*p* > 0.90). The odds ratio (OR) was 0.99 (95% CI: 0.52–1.84), indicating no significant difference in the likelihood of Foley catheter use between primiparous and multiparous women (Table 2).

### 3.3. Association Between Foley Catheter Use and Oral Misoprostol Dosing Regimen

The Foley catheter was used for additional mechanical cervical ripening in 91 of the 270 women included in the study. In crude analysis, Foley catheter placement appeared more frequent in women who received 50 µg of misoprostol compared with those who received 25 µg (78/210, 37.1% vs. 13/60, 21.7%). This association was statistically significant in the unadjusted analysis (Pearson’s chi-square test: χ^2^ = 4.9; *p* = 0.0374), with an odds ratio of 2.14 (95% CI: 1.10–4.15), indicating approximately twofold higher odds of Foley catheter use in the 50 µg group. However, in the subgroup analysis restricted to women without PROM, Foley catheter placement occurred in 13 of 35 cases (37.1%) in the 25 µg group and in 78 of 173 cases (45.1%) in the 50 µg group, and the difference was no longer statistically significant (OR = 1.39; 95% CI: 0.65–2.98; *p* = 0.39). After stratification by PROM status, no statistically significant difference in Foley catheter use between dosing groups was observed.

The time from the last misoprostol dose to delivery was shorter in the 50 µg group (median 291 min) compared with the 25 µg group (median 361 min); however, this difference did not reach statistical significance (*p* = 0.17). Although the interval from the last dose to delivery was analyzed based on available data, the interval from the first dose is generally considered a more clinically informative measure of total induction duration and should be evaluated in future studies.

The baseline characteristics and obstetric outcomes are presented in Table 3.

The distribution of parity did not differ significantly between the misoprostol dose groups. In the 25 µg group, 50 of 60 women (83.3%) were primiparous and 10 (16.7%) were multiparous, compared with 160 of 210 (76.2%) primiparous and 50 (23.8%) multiparous women in the 50 µg group. Pearson’s chi-square test showed no statistically significant difference in parity distribution between the groups (χ^2^ = 1.36; *p* = 0.3185), which was consistent with Fisher’s exact test (*p* > 0.05). The odds ratio (OR) was 1.56 (95% CI: 0.73–3.32).

The mode of delivery did not differ significantly between the misoprostol dose groups. In the 25 µg group, 48 of 60 women (80.0%) delivered vaginally and 12 (20.0%) underwent cesarean section, compared with 149 of 210 (71.0%) vaginal deliveries and 61 cesarean sections (29.0%) in the 50 µg group. Pearson’s chi-square test showed no statistically significant difference in delivery mode between the groups (χ^2^ = 1.97; *p* = 0.3027), which was confirmed by Fisher’s exact test (*p* > 0.05). The odds ratio (OR) for cesarean delivery was 1.64 (95% CI: 0.82–3.28).

### 3.4. Neonatal Outcomes

Neonatal outcomes were comparable between the two oral misoprostol dosing regimens (Table 4). Mean birthweight did not differ significantly between groups (3307 ± 450 g in the 25 µg group vs. 3370 ± 431 g in the 50 µg group; *p* = 0.33), confirming similar fetal maturity and prenatal growth at the time of delivery.

Immediate neonatal condition, assessed by Apgar scores, was generally very good in both groups. However, Apgar scores below 7 at 1 min were observed in 5 neonates (8.2%) in the 25 µg group and in 10 neonates (4.7%) in the 50 µg group. At 5 min, only one neonate in each group had an Apgar score below 7. These differences were not statistically significant.

Umbilical artery blood gas analysis demonstrated comparable neonatal acid–base status in both groups. Mean umbilical artery pH values were 7.30 ± 0.07 in the 25 µg group and 7.30 ± 0.09 in the 50 µg group (*p* = 0.80), while base excess values were −5.2 ± 3.2 and −5.6 ± 3.2, respectively (*p* = 0.35). No cases of clinically significant metabolic acidosis were identified.

Overall, the absence of significant differences in birthweight, Apgar scores (including the frequency of Apgar < 7), and umbilical cord blood gas parameters indicates that both oral misoprostol regimens have a comparable and favorable neonatal safety profile when used for labor preinduction in term pregnancies.

### 3.5. Safety Outcomes

Maternal and neonatal safety outcomes included uterine tachysystole, uterine hyperstimulation, need for tocolytic therapy, postpartum hemorrhage, non-reassuring cardiotocography, neonatal resuscitation, and admission to the neonatal intensive care unit (NICU). These outcomes were analyzed as a composite safety endpoint and additionally evaluated individually where data were available.

Uterine hyperstimulation occurred in three women (4.9%) in the 25 µg group and in ten women (4.7%) in the 50 µg group, with no statistically significant difference between groups (*p* = 1.0). No cases of neonatal resuscitation or NICU admission were recorded in either group. Furthermore, no cases of neonatal sepsis were documented.

Overall, no significant differences in the recorded maternal or neonatal safety outcomes were observed between the two dosing regimens.

## 4. Discussion

In this single-center retrospective cohort study, we compared two commonly used oral misoprostol preinduction regimens—25 µg administered every 2 h and 50 µg administered every 4 h—in term singleton pregnancies with an unfavorable cervix. Within the limits of the assessed outcomes, both regimens demonstrated comparable short-term maternal and neonatal results. The analysis focused on a composite maternal and neonatal safety outcome, allowing evaluation of clinically relevant adverse events while acknowledging the inherent limitations of retrospective data. Importantly, parity—but not maternal BMI—emerged as the key independent predictor of successful vaginal delivery following preinduction.

### 4.1. Neonatal and Maternal Safety

Both dosing strategies were associated with favorable neonatal outcomes. No statistically significant differences were observed in birthweight, Apgar scores at 1 and 5 min, or umbilical artery blood gas parameters between groups. Although Apgar scores below 7 at 1 min occurred slightly more frequently in the 50 µg group, this difference resolved by 5 min and was not clinically meaningful. No cases of clinically significant metabolic acidosis were identified.

Similarly, maternal safety endpoints—including rates of uterine tachysystole, hyperstimulation, and CTG abnormalities—were low and comparable between groups. These findings are consistent with previous randomized trials and systematic reviews indicating that low-dose oral misoprostol, when administered under standardized protocols and appropriate monitoring, is associated with a low incidence of fetal compromise [11,12,13,14]. Comparative studies evaluating misoprostol against other induction methods have likewise reported reassuring neonatal safety profiles [26].

The favorable safety profile of oral administration may partly be explained by its pharmacokinetic characteristics, including gradual absorption and lower peak plasma concentrations compared with other routes of administration, particularly vaginal use [25,27]. Our results further align with international recommendations supporting the use of oral misoprostol for cervical ripening at term, within the limits of the recorded safety outcomes and when applied within standardized clinical protocols [26].

However, in the absence of an external comparator group, our conclusions are limited to relative comparisons between the two regimens and do not establish absolute safety beyond the measured outcomes.

### 4.2. Dosing Strategy, Foley Catheter Use, and the Role of PROM

In the crude analysis, the need for additional mechanical cervical ripening with a Foley catheter was significantly higher in the 50 µg group. At first glance, this finding could suggest reduced pharmacologic effectiveness of the higher-dose regimen. However, further analyses provided important contextual clarification.

PROM was substantially more frequent in the 25 µg group, and since membrane status may influence cervical favorability and subsequent clinical decision-making, this imbalance represents a potential source of confounding by indication. When the analysis was restricted to women without PROM, the difference in Foley catheter use between dosing groups was no longer statistically significant.

The attenuation of the observed effect after stratification suggests that the initially detected difference in Foley catheter utilization may not represent a true dose-dependent pharmacologic effect but rather differences in baseline obstetric characteristics influencing clinical management pathways. In clinical practice, the presence or absence of PROM may modify both the perceived urgency of induction and the threshold for escalation to mechanical methods.

Similar patterns have been described in studies comparing pharmacologic and mechanical cervical ripening strategies, where escalation to mechanical methods often reflects cervical resistance rather than primary drug inefficacy [8,9,28]. Furthermore, PROM has been shown to influence induction response and subsequent management decisions [26,29].

Thus, the observed association between higher Foley catheter use and the 50 µg regimen should be interpreted cautiously, particularly in light of the imbalance in membrane status between groups.

### 4.3. Subsequent Management After Foley Catheter Placement

Among women who required mechanical cervical ripening, a heterogeneous pattern of subsequent labor progression was observed. A proportion developed spontaneous uterine contractions without the need for additional pharmacologic augmentation, whereas others progressed to advanced cervical dilation following Foley catheter removal and subsequently underwent artificial rupture of membranes. In the remaining cases, labor augmentation with oxytocin was required.

These findings suggest that Foley catheter placement should not be uniformly interpreted as failed pharmacologic preinduction, but rather as a transitional step within a multimodal induction pathway. The variability in subsequent management underscores the complexity of induction dynamics and supports cautious interpretation of Foley catheter use as an isolated outcome marker.

### 4.4. Role of Parity in Preinduction Success

Parity emerged as the strongest independent predictor of successful vaginal delivery in our cohort. Multiparous women had approximately 2.5-fold higher odds of achieving vaginal birth compared with nulliparous women. This finding is consistent with well-established obstetric evidence identifying nulliparity as one of the most robust predictors of prolonged induction and increased risk of cesarean delivery following induction of labor [13,20].

Importantly, this association remained significant independently of the misoprostol dosing regimen, suggesting that inherent differences in cervical and uterine responsiveness related to parity may play a greater role in induction success than modest variations in pharmacologic dosing schedules.

### 4.5. Maternal BMI and Induction Outcomes

Although the 50 µg group had a significantly higher median BMI, no clear association between maternal BMI and the analyzed obstetric outcomes was observed in the available univariable analyses. However, given the retrospective design of the study and baseline differences between groups, residual confounding related to BMI cannot be excluded.

These observations are consistent with previous studies suggesting that, although obesity may affect labor dynamics and prolong labor duration, it does not necessarily lead to worse obstetric or neonatal outcomes when low-dose oral misoprostol is used within standardized clinical protocols [21,22,23].

Within the limitations of this retrospective cohort, our findings suggest that maternal BMI alone may not substantially modify the short-term obstetric outcomes associated with oral misoprostol preinduction.

### 4.6. Clinical Implications

Taken together, our findings suggest that both oral misoprostol regimens are associated with comparable short-term maternal and neonatal outcomes within the analyzed parameters. Differences observed in the need for mechanical cervical ripening appear to be influenced, at least in part, by baseline clinical characteristics such as PROM rather than by dosing regimen alone.

From a clinical perspective, both dosing strategies may be considered acceptable when applied within standardized institutional protocols. Parity, which emerged as an independent predictor in our analysis, should be taken into account during patient counseling, as expectations regarding induction success and the likelihood of cesarean delivery may differ substantially between nulliparous and multiparous women. In contrast, maternal BMI alone does not appear to warrant modification of the preinduction strategy based on the analyzed outcomes.

From a practical standpoint, the 25 µg regimen may be preferred in patients with PROM, where cervical favorability is often more advanced and excessive uterine activity should be minimized. The 50 µg regimen may remain appropriate in selected patients requiring stronger pharmacologic stimulation, although clinicians should be aware of a potentially higher likelihood of escalation to mechanical cervical ripening. Additionally, the choice of regimen may influence workflow, as the 25 µg schedule requires more frequent administration and monitoring, potentially increasing staff workload while allowing finer dose titration.

### 4.7. Strengths and Limitations

This retrospective observational study was conducted and reported in accordance with the RECORD statement for observational research using routinely collected health data [25]. The strengths of this study include the use of routinely collected clinical data, the implementation of standardized institutional management protocols, and the comprehensive assessment of both obstetric and neonatal outcomes.

Nevertheless, several limitations should be acknowledged. The retrospective design and non-randomized allocation of dosing regimens introduce the possibility of residual confounding. In particular, the imbalance in PROM distribution between groups may have influenced subsequent management decisions and limited causal inference regarding potential dose-related effects. Furthermore, the single-center setting may restrict the generalizability of the findings.

An additional methodological limitation is that the duration of induction was assessed using the interval from the last administered dose rather than from the first dose of misoprostol, which may more accurately reflect total induction time. Furthermore, failed induction was defined based on completion of the institutional stepwise induction protocol rather than a single predefined time threshold, which may limit comparability with studies using fixed time criteria. Moreover, certain maternal and neonatal safety parameters suggested in the previous literature—such as placental abruption, chorioamnionitis, meconium-stained amniotic fluid, and maternal analgesic requirements—were not systematically recorded in all cases and therefore could not be included in the primary analysis. As a result, conclusions regarding safety are limited to the outcomes consistently documented in the medical records.

Prospective randomized studies stratified by membrane status and parity are warranted to further clarify the optimal dosing strategy for oral misoprostol preinduction and to provide more definitive conclusions regarding induction dynamics and safety outcomes.

## Figures and Tables

**Table 1:** Multivariable logistic regression analysis for vaginal delivery.

Variable	Adjusted OR	95% CI	*p*-Value
50 µg misoprostol dose	1.21	0.68–2.16	0.51
Multiparity	2.51	1.17–5.39	0.018
BMI	0.98	0.94–1.02	0.32
PROM	1.09	0.61–1.95	0.77
Foley catheter use	0.84	0.46–1.52	0.57

**Table 2:** Comparison of Foley catheter use between primiparous and multiparous women.

Parity	Foley Catheter	
	Yes	No
Multiparity	20 (33.3%)	40 (66.7%)
Nulliparity	71 (33.8%)	141 (66.2%)

*p*-value = 0.8948.

**Table 3:** Comparison between patients who underwent labor preinduction with oral misoprostol at a dose of 25 micrograms versus 50 micrograms.

		Group 25 µg, *n* = 60	Group 50 µg, *n* = 210	*p*-Value
Maternal age [years]	Mean (±SD)	30 years (±4)	31 years (±5)	0.7011
Gestation age at delivery [weeks]	Median (Range)	39 (38–41)	39 (38–42)	0.1478
BMI	Median (Range)	27 (17–48)	31 (21–62)	0.0001
Nulliparity		50 (83.3%)	160 (76.2%)	0.3185
Multiparity		10 (16.7%)	50 (23.8%)	
Spontaneous delivery		48 (80.0%)	149 (71.0%)	0.3027
Cesarean section		13 (20.0%)	61 (29.0%)	
Time from administration the last dose to delivery [min.]	Median (Range)	361 (147–933)	291 (38–1092)	0.1754
Number of doses	Median (Range)	6 (1–8)	4 (1–4)	<0.0001
Total dose of misoprostol [μg]	Median (Range)	150 (25–200)	200 (50–200)	<0.0001
Indication for preinduction				
Diabetes		13 (23%)	70 (33.7%)	0.1167
Hypertension		1 (1.6%)	15 (7.1%)	0.1970
Diabetes + Hypertension		1 (1.6%)	13 (6.2%)	0.2807
Preeclampsia		1 (1.6%)	0	NA
PROM		25 (41%)	37 (17.5%)	0.0002
FGR		4 (6.6%)	10 (4.7%)	0.8126
Post-term pregnancy		4 (6.6%)	12 (5.7%)	0.9565
Others		11 (18%)	53 (25.1%)	0.3282

**Table 4:** Neonatal outcome.

	Group 25 µg	Group 50 µg	*p*-Value
Birthweight [g] mean (±SD)	3307 (±450)	3370 (±431)	0.3276
Apgar 1 Median (Range)	10 (1–10)	10 (2–10)	0.0720
Apgar 5Median (Range)	10 (1–10)	10 (6–10)	0.3457
PH mean (±SD)	7.30 (±0.07)	7.30 (±0.09)	0.7959
BE	−5.2 (±3.2)	−5.6 (±3.2)	0.3547

## Data Availability

The data presented in this study are available on request from the corresponding author. The data are not publicly available due to privacy and ethical restrictions related to patient confidentiality.

## Abbreviations

The following abbreviations are used in this manuscript:

BMI	Body mass index
CTG	Cardiotocography
PROM	Prelabour rupture of membranes
FGR	Fetal growth restriction
